# Urinary Phthalate Metabolites and Biomarkers of Oxidative Stress in Pregnant Women: A Repeated Measures Analysis

**DOI:** 10.1289/ehp.1307996

**Published:** 2014-11-14

**Authors:** Kelly K. Ferguson, Thomas F. McElrath, Yin-Hsiu Chen, Bhramar Mukherjee, John D. Meeker

**Affiliations:** 1 Department of Environmental Health Sciences, University of Michigan School of Public Health, Ann Arbor, Michigan, USA; 2 Division of Maternal-Fetal Medicine, Brigham and Women’s Hospital, Harvard Medical School, Boston, Massachusetts, USA; 3 Department of Biostatistics, University of Michigan School of Public Health, Ann Arbor, Michigan, USA

## Abstract

**Background:**

Phthalate exposure occurs readily in the environment and has been associated with an array of health end points, including adverse birth outcomes. Some of these may be mediated by oxidative stress, a proposed mechanism for phthalate action.

**Objectives:**

In the present study, we explored the associations between phthalate metabolites and biomarkers of oxidative stress measured in urine samples from multiple time points during pregnancy.

**Methods:**

Women were participants in a nested case–control study of preterm birth (*n* = 130 cases, *n* = 352 controls). Each was recruited early in pregnancy and followed until delivery, providing urine samples at up to four visits. Nine phthalate metabolites were measured to assess exposure, and 8-hydroxydeoxyguanosine and 8-isoprostane were also measured in urine as markers of oxidative stress. Associations were assessed using linear mixed models to account for intraindividual correlation, with inverse selection probability weightings based on case status to allow for greater generalizability.

**Results:**

Interquartile range increases in phthalate metabolites were associated with significantly higher concentrations of both biomarkers. Estimated differences were greater in association with monobenzyl phthalate (MBzP), mono-*n*-butyl phthalate (MBP), and monoisobutyl phthalate (MiBP), compared with di(2-ethylhexyl) phthalate (DEHP) metabolites.

**Conclusions:**

Urinary phthalate metabolites were associated with increased oxidative stress biomarkers in our study population of pregnant women. These relationships may be particularly relevant to the study of birth outcomes linked to phthalate exposure. Although replication is necessary in other populations, these results may also be of great importance for a range of other health outcomes associated with phthalates.

**Citation:**

Ferguson KK, McElrath TF, Chen YH, Mukherjee B, Meeker JD. 2015. Urinary phthalate metabolites and biomarkers of oxidative stress in pregnant women: a repeated measures analysis. Environ Health Perspect 123:210–216; http://dx.doi.org/10.1289/ehp.1307996

## Introduction

Phthalate diesters are used as plasticizers and solvents in a variety of consumer products, and can readily enter human systems through ingestion, inhalation, and dermal absorption (Agency for Toxic Substances and Disease Registry 2001, 2002). Although diesters are metabolized and excreted quickly, constant contact results in daily exposures for most of the U.S. population. Metabolites are consistently detected in urine of pregnant women in populations worldwide (Cantonwine et al. 2013; Lin et al. 2011; Woodruff et al. 2011; Zeman et al. 2013).

Although phthalates are best known for their action as endocrine disruptors, there is also evidence from *in vitro* and animal studies that mono(2-ethylhexyl) phthalate (MEHP) may cause oxidative stress by inducing release of reactive oxygen species (ROS) and/or impairing antioxidant defenses (Erkekoglu et al. 2010; Kasahara et al. 2002; Tetz et al. 2013; Zhao et al. 2012). However, few studies have examined this association in humans. Three cross-sectional studies have observed associations between some phthalate metabolites and serum levels of bilirubin, a potent antioxidant, and systemic markers of oxidative stress including serum gammaglutamyl transferase, and urinary malondialdehyde (MDA) and 8-hydroxydeoxyguanosine (8-OHdG) (Ferguson et al. 2011, 2012; Hong et al. 2009). A more recent study in elderly subjects also observed an association between summed di(2-ethylhexyl) phthalate (DEHP) metabolites and MDA (Kim et al. 2013).

To our knowledge, no studies have examined the relationship between urinary phthalate metabolites and biomarkers of oxidative stress during pregnancy, when these possible effects represent particular concern given the gestational vulnerability of the developing fetus. Increases in oxidative stress biomarkers in pregnant women have been associated with pregnancy loss, preeclampsia, preterm birth, and fetal growth restriction (Agarwal et al. 2012; Stein et al. 2008). Additionally, the potential effects of phthalate exposures on oxidative stress are relevant to a number of other outcomes in the general population, such as infertility, various cancers, and type 2 diabetes. In the present study, we examined associations between repeated measures of urinary phthalate metabolites and biomarkers of oxidative stress in pregnant women.

## Methods

*Study population*. Pregnant women were recruited before 15 weeks gestation at Brigham and Women’s Hospital in Boston, Massachusetts, from 2006 through 2008 as part of a large prospective cohort study and provided informed consent upon enrollment. Participants were included if they had a singleton pregnancy that resulted in a live birth. Women were followed throughout the duration of pregnancy and provided demographic and anthropometric data, urine samples from up to four study visits (targeted for 10, 18, 26, and 35 weeks gestation), as well as birth outcome data at delivery. Gestational age was calculated from last menstrual period and validated with first-trimester ultrasound; if gestational ages calculated by the two methods differed by > 8%, ultrasound dating was used. For the present study, 130 women who delivered preterm and 352 random controls were selected, and their urine samples were extracted from –80°C storage for laboratory analysis (*n* = 482 subjects total). Institutional review board approval for this study was obtained from the University of Michigan and Brigham and Women’s Hospital.

The nested case–control study was designed with the intention of examining associations between urinary phthalate metabolites across gestation and preterm birth (Ferguson et al. 2014b). The present analysis examining the relationship between urinary phthalate metabolites and biomarkers of oxidative stress was a secondary aim of this study to help inform potential biological mechanisms involved. Our goal was to characterize these associations in a population that would be generalizable to the overall cohort. Therefore, the analyses were weighted using inverse probability weightings calculated based on selection probabilities for cases (90.1%) and controls (33.9%) from the parent cohort population (Jiang et al. 2006).

*Urinary phthalate metabolites*. All available urine samples (*n* = 1,693) were assayed for concentrations of nine phthalate metabolites using high-performance liquid chromatography and tandem mass spectrometry by NSF International in Ann Arbor, Michigan (Lewis et al. 2013; Silva et al. 2007). All metabolites were highly detectable (> 95%); levels below the limit of detection (LOD) were replaced with the LOD divided by the square root of 2 (Hornung and Reed 1990). All distributions were right-skewed and natural log-transformed to meet normality assumptions for statistical analysis. To adjust for urine dilution, specific gravity (SG) was measured at the time of phthalate analysis using a handheld refractrometer (Atago Co., Ltd., Tokyo, Japan). For bivariate analysis we created SG-corrected concentrations using the following formula: *P_c_* = *P*[(1.015 – 1)/(*SG* – 1)], where *P_c_* is the corrected phthalate concentration, *P* is the raw concentration, 1.015 is the median SG for the study population, and *SG* is the specific gravity for the sample (Meeker et al. 2009). In regression models uncorrected phthalate metabolite levels were used and models were adjusted for SG as a covariate.

In addition to examining individual urinary phthalate metabolites, we created a summed measure of DEHP metabolites (ΣDEHP). MEHP, mono(2-ethyl-5-hydroxyhexyl) phthalate (MEHHP), mono(2-ethyl-5-oxohexyl) phthalate (MEOHP), and mono(2-ethyl-5-carboxypentyl) phthalate (MECPP) concentrations were converted from micrograms per liter to micromoles per liter using molecular weights (278, 294, 292, and 308 g/mol) and summed to create ΣDEHP.

*Oxidative stress biomarkers*. All urine samples with sufficient volume remaining following phthalate measurement (*n* = 1,678) were assayed for levels of 8-OHdG and total 8-isoprostane using enzyme immunoassay by Cayman Chemical (Ann Arbor, MI). For 8-isoprostane measurement only, samples were first hydrolyzed and passed through columns for affinity purification. LODs were 3.9 pg/mL and 10.3 pg/mL for urine 8-isoprostane and 8-OHdG concentrations, respectively. As with phthalate metabolites, undetected oxidative stress measures were replaced with the LOD divided by the square root of 2 (Hornung and Reed 1990). For calculation of distributions overall and by categorical covariates biomarkers were corrected for urinary SG using the formula applied to phthalate measures above. Uncorrected biomarker concentrations were used for multivariate models with SG as a covariate.

*Statistical analysis*. All statistical analysis was performed in R version 2.15.2 (R Foundation for Statistical Computing, Vienna, Austria) and SAS version 9.2 (SAS Institute Inc., Cary, NC). Unless stated otherwise, all analysis was performed with inverse probability weightings. Overall distributions of urinary phthalate metabolites and oxidative stress biomarkers were assessed using SG-corrected concentrations. Variability in oxidative stress biomarker concentrations across gestation was examined using intraclass correlation coefficients (ICCs), which represent a ratio of within to between individual variability (Rosner 2011). ICCs for urinary phthalate metabolites have been examined previously (unpublished data), and were similar to other studies observing greater reproducibility of monobenzyl phthalate [MBzP; ICC = 0.61; 95% confidence interval (CI) = 0.56–0.65], mono-*n*-butyl phthalate (MBP; ICC = 0.57; 95% CI: 0.53, 0.62), and monoisobutyl phthalate (MiBP; ICC = 0.52, 95% CI: 0.48, 0.57) compared with DEHP metabolites (ICC range, 0.19–0.31) across pregnancy (Adibi et al. 2008; Braun et al. 2012). Geometric means and standard deviations of corrected concentrations were created by categorical covariate groups and compared using linear mixed models (LMMs) with subject-specific random intercepts to adjust for intraindividual correlation of measurements at multiple time points (*nlme* package in R) (Pinheiro et al. 2013). Covariates examined included race/ethnicity (white, African American, or other), education level (high school, technical school, some college or junior college, college graduate or above), health insurance provider (public or private), body mass index (BMI) at the initial visit (continuous), tobacco and alcohol use during pregnancy (yes or no), parity (nulliparous or parous), sex of fetus (male or female), and use of assisted reproductive technology (yes or no). We also examined time-varying covariates, including BMI (continuous) and time of day of urine sample collection (dichotomized into before versus after 1300 hours based on histograms which displayed a nadir in urinary phthalate metabolite concentrations at that time of day).

Associations (fixed effects) between uncorrected urinary phthalate metabolites and oxidative stress biomarkers were estimated using LMM with subject-specific random intercepts to adjust for intraindividual correlation (random slopes did not improve model fit based on the Akaike information criterion). For all statistical models very concentrated urine samples (SG > 1.04) were excluded because biomarkers measured in those samples may be inaccurate (*n* = 4) (Boeniger et al. 1993; Braun et al. 2012). One oxidative stress biomarker was regressed on one phthalate metabolite per model. Crude models were adjusted for gestational age and urinary SG. Full models additionally included covariates that were significantly (*p* < 0.05) associated with one or both oxidative stress biomarkers as well as one or more urinary phthalate metabolites. Final full models were adjusted for urinary specific gravity, gestational age at sample collection, race/ethnicity, education level, health insurance provider, BMI, time of day of urine sample collection, and parity of infant. Tobacco and alcohol use were excluded from adjusted models because of the small number of subjects who used either during pregnancy (*n* = 31 and 20, respectively). Subjects with missing visit 1 covariates were excluded from LMM models; if time-varying covariates were missing, then individual time points only (not all data for that subject) were excluded. Statistical significance of effect estimates was assessed with an alpha level of 0.05.

Several sensitivity analyses were performed. First we examined associations in a stratified analysis of cases and controls separately. Second, we examined associations after excluding mothers who used alcohol and tobacco during pregnancy, because the number of users was too small to include these as covariates. Third, we created generalized additive mixed models (GAMMs) to investigate the possibility that the relationships between oxidative stress biomarkers and urinary phthalate metabolites were nonlinear. Fourth, to examine whether the relationship between phthalate exposure and oxidative stress differed based on time point in pregnancy, we examined interaction terms between urinary metabolites and either study visit or gestational age at sample collection, also in LMMs with random intercepts. These models were created in preterm cases and controls separately, because of the difference in proportions of cases compared with controls with measurements available at each time point, particularly at visit 4. Finally, we estimated associations based on LMM models adjusted for multiple urinary phthalate metabolites. We selected metabolites for multiple metabolite models based on their correlations with one another as well as their individual associations with the oxidative stress biomarkers.

## Results

Population demographics have been presented elsewhere for the case–control study population (Ferguson et al. 2014b) and are presented in Table 1 for the weighted population examined in the present analysis. Demographics for the population with complete covariates for regression models were almost identical and are presented in Supplemental Material, Table S1. Maternal age at visit 1 was 32.0 years on average, and most women were white (59%), well educated (71% with junior college, some college, or above), did not use tobacco (94%) or alcohol (95%) during pregnancy (Table 1), and did not use assisted reproductive technology to get pregnant (91%). More than half of infants were female (55%). Approximately half of the participants had normal to low BMI at visit 1 (53%) (Table 1), and as expected this proportion decreased across pregnancy (visit 2 = 47%, visit 3 = 28%, visit 4 = 15%). After weighting, deliveries in the study population were approximately 12% preterm (< 37 weeks gestation).

**Table 1:** Demographic characteristics in weighted study population (*n* = 482).

Characteristic	Percent
Race/ethnicity (*n *= 482)
White	59
African American	16
Other	26
Education (*n *= 471)
High school	14
Technical school	16
Junior college or some college	30
College graduate	41
Health insurance (*n *= 470)
Private/HMO/self-pay	81
Medicaid/SSI/MassHealth	19
Body mass index at visit 1 (*n *= 478)
< 25 kg/m^2^ (underweight to normal)	53
25–30 kg/m^2^ (overweight)	27
≥ 30 kg/m^2^ (obese to morbidly obese)	20
Smoking during pregnancy (*n *= 476)
Some	6
None	94
Alcohol use during pregnancy (*n *= 472)
Some	5
None	95
Parity (*n *= 482)
Nulliparous	45
Parous	55
Abbreviations: HMO, health maintenance organization; MassHealth, Massachusetts state health insurance provider; SSI, supplemental security income. Distributions of demographic characteristics were created from inverse probability weightings for case–control status.	

Urine samples were collected from study participants at up to four visits (mean 3.52 samples per subject; *n* = 3 subjects with one urine sample; *n* = 38 subjects with two samples; *n* = 148 subjects with three samples; *n* = 293 subjects with four samples). Samples for visit 1 (median 9.95 weeks gestation) were collected for 474 subjects (range, 4.17–19.1 weeks); for visit 2 (median, 18.0 weeks) samples were collected for 421 subjects (range, 14.9–32.1 weeks); for visit 3 (median, 26.0 weeks) samples were collected for 409 subjects (range, 22.9–36.3 weeks); and for visit 4 (median, 35.2 weeks) samples were collected for 374 subjects (range, 33.1–38.3 weeks). The proportion of cases with samples available was consistent for visits 1–3 (86–100%) but low for visit 4 (51%) because many had already delivered by that time point. Most urine samples were collected before 1300 hours at visits 1 (60%), 2 (66%), 3 (70%), and 4 (69%).

Urinary phthalate metabolite and oxidative stress biomarker distributions are presented in Table 2. Phthalate metabolites were detected in 95–100% of all samples measured, 8-OHdG was detected in all samples, and 8-isoprostane was below the LOD in 67 (4.0%) samples. As reported previously, correlations between phthalate metabolites were strongest within DEHP metabolites, as expected (Spearman *r* = 0.68–0.91), were moderate between MBzP, MBP, and MiBP (*r* = 0.46–0.62) and between mono(3-carboxypropyl) phthalate (MCPP) and DEHP metabolites (*r* = 0.35–0.46), and were weak between all other metabolites (*r* = 0.01–0.21) (Ferguson et al. 2014a). Correlations between the two oxidative stress markers at each visit were weak but statistically significant (*r* = 0.10–0.20, *p* < 0.05). 8-OHdG concentrations were more variable across pregnancy (ICC = 0.32; 95% CI: 0.27, 0.38) compared with 8-isoprostane (ICC = 0.60; 95% CI: 0.56, 0.64).

**Table 2:** Distributions of phthalate metabolites and oxidative stress biomarkers measured in urine samples collected from up to four time points during pregnancy in all samples measured from weighted population.

Biomarker	LOD	% < LOD	Geometric mean (geometric SD)	Percentile					
				25th	50th	75th	90th	95th	Maximum
MEHP (μg/L)	1.0	4.7	10.6 (3.49)	4.63	9.07	21.0	56.3	106	1,555
MEHHP (μg/L)	0.1	0.0	34.2 (3.41)	14.9	27.5	70.3	182	305	2,850
MEOHP (μg/L)	0.1	0.1	18.3 (3.32)	8.33	15.3	37.6	93.0	152	1,128
MECPP (μg/L)	0.2	0.0	43.5 (3.40)	17.7	34.9	99.4	231	391	3,713
∑DEHP (μmol/L)			0.39 (3.17)	0.17	0.31	0.78	2.00	3.01	21.1
MBzP (μg/L)	0.2	1.1	7.07 (3.05)	3.47	6.38	13.2	29.0	55.8	465
MBP (μg/L)	0.5	0.3	17.8 (2.40)	10.9	16.5	27.8	46.0	61.5	24,879
MiBP (μg/L)	0.1	0.4	7.61 (2.29)	4.74	7.57	12.0	19.6	27.4	351
MEP (μg/L)	1.0	0.1	141 (4.68)	47.3	121	383	1,084	2,307	48,130
MCPP (μg/L)	0.2	3.2	2.10 (3.14)	1.03	1.68	3.50	8.60	19.6	848
8-OHdG (ng/mL)	0.01	0.0	130 (1.66)	98.4	130	173	233	288	1,339
8-Isoprostane (pg/mL)	3.9	4.0	180 (2.64)	130	210	320	460	574	2,784
Abbreviations: 8-OHdG, 8-hydroxydeoxyguanosine; LOD, limit of detection. All biomarkers were corrected for urinary specific gravity. For urinary phthalate metabolites, *n *= 1,693 samples, 482 subjects; for urinary oxidative stress biomarkers, *n *= 1,678 samples, 482 subjects.									

When oxidative stress biomarker concentrations were compared by categorical covariates, we observed different patterns for each marker (Table 3). Levels of 8-isoprostane were lowest in mothers who were white, had higher levels of education and private health insurance, were of lower BMI at visit 1, did not use tobacco or alcohol, and were parous. Few differences were observed by categorical covariates for 8-OHdG, although mothers with private health insurance had significantly lower levels. Associations with covariates were also different for urinary phthalate metabolites. MEHP concentrations (Table 3) and other DEHP metabolites (see Supplemental Material, Table S2) were higher in African-American compared with white mothers but no other differences were observed. Patterns for MBzP, MiBP, and monoethyl phthalate (MEP) (see Supplemental Material, Table S2) were similar to those for MBP (Table 3); higher concentrations were observed in mothers who were African American or other race/ethnicity compared with white, in mothers with lower education levels, in mothers with public compared with private health insurance, and in mothers with higher BMI at visit 1. No statistically significant differences in oxidative stress biomarkers and few differences in urinary phthalate metabolites were observed by fetus sex (lower MiBP concentrations in mothers of female vs. male fetus) or use of assisted reproductive technology (higher MEHHP concentrations and lower MBzP and MiBP concentrations in mothers who used assisted reproductive technology compared with those who did not) (data not shown).

**Table 3:** Oxidative stress and exposure biomarker concentrations (geometric mean and geometric standard deviation) by categorical demographic characteristics in all samples measured from weighted population.

Characteristic	8-OHdG (ng/mL)	8-Iso (pg/mL)	MEHP (μg/L)	MBP (μg/L)
Race/ethnicity
White (reference)	130 (1.14)	153 (1.69)	10.1 (2.20)	15.4 (1.37)
African American	133 (1.11)	277 (1.33)*	13.5 (2.34)*	23.6 (1.45)*
Other	129 (1.15)	205 (1.43)*	10.4 (2.02)	21.2 (1.63)*
Education
High school (reference)	146 (1.11)	289 (1.41)	9.20 (2.11)	27.8 (1.29)
Technical school	133 (1.11)	217 (1.51)	9.91 (2.13)	19.6 (1.48)*
Junior college or some college	124 (1.17)*	173 (1.65)*	9.57 (2.24)	16.7 (1.62)*
College graduate	128 (1.13)	143 (1.57)*	12.0 (2.11)	15.6 (1.37)*
Health insurance
Private insurance/HMO/self-pay (reference)	126 (1.14)	162 (1.64)	10.6 (2.19)	16.0 (1.47)
Medicaid/SSI/MassHealth	151 (1.09)*	271 (1.33)*	9.74 (2.05)	29.1 (1.28)*
BMI at visit 1
< 25 kg/m^2^ (reference)	128 (1.13)	155 (1.64)	10.8 (2.19)	16.7 (1.37)
25 to < 30 kg/m^2^	132 (1.12)	181 (1.59)	10.5 (2.27)	16.5 (1.54)
≥ 30 kg/m^2^	136 (1.17)	270 (1.34)*	10.6 (2.10)	23.6 (1.56)*
Tobacco use
Smoked during pregnancy (reference)	150 (1.11)	320 (1.47)	9.87 (1.83)	26.4 (1.34)
No smoking during pregnancy	129 (1.14)	173 (1.60)*	10.6 (2.19)	17.5 (1.47)*
Alcohol use
Alcohol use during pregnancy (reference)	138 (1.06)	201 (1.29)	12.3 (2.27)	15.0 (1.19)
No alcohol use during pregnancy	130 (1.14)	178 (1.63)	10.5 (2.16)	18.1 (1.49)
Parity
Nulliparous (reference)	129 (1.13)	166 (1.57)	11.4 (2.23)	17.0 (1.39)
Parous	131 (1.14)	192 (1.62)*	10.1 (2.14)	18.6 (1.53)
Abbreviations: 8-Iso, 8-isoprostane; 8-OHdG, 8-hydroxydeoxyguanosine; HMO, health maintenance organization; MassHealth, Massachusetts state health insurance provider; SSI, supplemental security income. All biomarkers were corrected for urinary specific gravity. For urinary phthalate metabolites, *n *= 1,693 samples, 482 subjects; for urinary oxidative stress biomarkers, *n *= 1,678 samples, 482 subjects. *p < 0.05 for significant difference in biomarker concentration from reference category, estimated from weighted linear mixed model with random intercepts for subject identification.				

For time-varying covariates, significantly higher oxidative biomarkers concentrations were observed in samples collected before versus after 1300 hours, and significantly lower urinary phthalate metabolite concentrations were observed in samples collected before versus after 1300 hours for all metabolites except MiBP and MEP, which were slightly higher in the morning (data not shown). When BMI was examined as a time-varying covariate, both oxidative stress biomarkers were positively associated with increasing BMI category (data not shown). MEHP was inversely associated with the highest BMI category, and MBzP was positively associated with the highest BMI category, but otherwise associations with urinary phthalate metabolites were close to the null (data not shown). Because BMI measures at each study visit may more accurately capture confounding by this variable, time-varying BMI was included as a covariate in fully adjusted models.

Effect estimates from adjusted models were similar to those from crude models (data not shown); adjusted results alone are presented in Table 4 (*n* = 464 subjects with complete data). Fixed-effect results are presented in the form of percent change in oxidative stress biomarker with an interquartile range (IQR) increase in untransformed phthalate metabolite. All phthalate metabolites were associated with higher 8-OHdG concentrations; the largest percent changes with an IQR increase in exposure observed were for MBzP (20.7%; 95% CI: 15.6, 26.1%), MBP (18.1%; 95% CI: 13.5, 22.9%), and MiBP (30.3%; 95% CI: 24.4, 36.5%). All metabolites were associated with significantly higher 8-isoprostane concentrations, and coefficients were larger compared with those estimated for 8-OHdG. However, as with 8-OHdG, the largest differences in 8-isoprostane were in association with MBzP (42.7%; 95% CI: 31.8, 54.4%), MBP (42.0%; 95% CI: 32.0, 52.7%), and MiBP (56.4%; 95% CI: 43.9, 69.9%).

**Table 4:** Percent difference (95% CIs) in oxidative stress biomarker in association with IQR increase in phthalate metabolite level.

Metabolite	IQR^*a*^	8-OHdG		8-Isoprostane	
		% difference (95% CI)	*p*-Value	% difference (95% CI)	*p*-Value
MEHP	16.6 μg/L	2.74 (–0.47, 6.05)	0.09	14.1 (8.06, 20.5)	< 0.001
MEHHP	56.9 μg/L	8.40 (4.93, 12.0)	< 0.001	15.8 (9.53, 22.4)	< 0.001
MEOHP	29.4 μg/L	7.34 (4.01, 10.8)	< 0.001	15.9 (9.87, 22.3)	< 0.001
MECPP	80.5 μg/L	6.53 (2.96, 10.2)	< 0.001	23.0 (16.0, 30.4)	< 0.001
∑DEHP	0.63 μmol/L	6.67 (3.23, 10.2)	< 0.001	19.1 (12.7, 25.9)	< 0.001
MBzP	12.5 μg/L	20.7 (15.6, 26.1)	< 0.001	42.7 (31.8, 54.4)	< 0.001
MBP	24.8 μg/L	18.1 (13.5, 22.9)	< 0.001	42.0 (32.0, 52.7)	< 0.001
MiBP	11.3 μg/L	30.3 (24.4, 36.5)	< 0.001	56.4 (43.9, 69.9)	< 0.001
MEP	355 μg/L	11.5 (7.32, 15.9)	< 0.001	19.7 (11.8, 28.2)	< 0.001
MCPP	2.98 μg/L	7.23 (3.83, 10.7)	< 0.001	20.2 (13.7, 27.1)	< 0.001
8-OHdG, 8-hydroxydeoxyguanosine. Estimates were from adjusted linear mixed-effect models with random intercepts for subject identification (*n* = 1,604 samples, *n* = 464 subjects). The models were adjusted for urinary specific gravity, gestational age at sample collection, race/ethnicity, education level, health insurance provider, BMI (time-varying), time of day of urine sample collection (before vs. after 1300 hours, time-varying), and parity of infant. Models include inverse probability weights to adjust for case–control study design. ^***a***^Ranges differ slightly from 25th–75th percentile differences in Table 2 because they were calculated from raw phthalate metabolite concentrations, uncorrected for urinary specific gravity.					

*Sensitivity analyses*. LMM were replicated when stratifying by preterm birth case status. Effect estimates were similar with some exceptions (Table S3). In cases alone, associations between 8-OHdG and MBP (8.92%; 95% CI: 2.03, 16.3%), MiBP (20.3%; 95% CI: 10.2, 31.3%), and MEP (19.2%; 95% CI: 10.3, 28.8%) and also between 8-isoprostane and MBP (30.9%; 95% CI: 15.8, 48.0%) were smaller than corresponding associations estimated using weighted models. Effect estimates from models of controls alone were similar to those from weighted models. Effect estimates from weighted models will be closer to control rather than case estimates from Supplemental Material, Table S3, because there are many more controls than cases in the weighted population. Also, the effect estimates from the weighted models do not necessarily fall between case and control effect estimates because of differences in IQR ranges for cases compared with controls as well as differences in covariate distributions and association with exposure/outcome within case and control groups.

We also examined the effect of removing alcohol and tobacco users from the population; model estimates were similar to those for the overall population (data not shown). GAMM models were created to examine deviation of the oxidative stress–phthalate metabolite relationships from linearity. Models included the same covariates as in LMM. Based on visual inspection of smooth plots, relationships were linear for most metabolites and for those that were not, smooth curves deviated minimally from linearity (data not shown).

To identify potentially sensitive time points for the relationship between phthalate exposure and maternal oxidative stress, we additionally examined interaction terms in fully adjusted LMM between phthalate metabolite and gestational age at sample collection or visit of sample collection. No significant (*p* < 0.05) interactions with gestational age or study visit were observed for 8-OHdG or 8-isoprostane models (data not shown).

Finally, we examined the effect of including multiple phthalate metabolites in the same model. First we examined ΣDEHP metabolites with MBP, because these measures were weakly correlated and would not create problems with multicollinearity (Chatterjee et al. 2000; Ferguson et al. 2014a), were strong predictors of oxidative stress in the present analysis, and previous animal and *in vitro* research on phthalate-induced oxidative stress has focused on the parent compounds of these two metabolites, DEHP (Erkekoglu et al. 2010; Kasahara et al. 2002; Rusyn et al. 2001) and dibutyl phthalate (DBP) (Kim et al. 2002; Shono and Taguchi 2014; Zhou et al. 2010). In the model for 8-OHdG, the effect estimate was lower for ΣDEHP (4.32%; 95% CI: 0.98, 7.78%) and very similar for MBP (17.0%; 95% CI: 12.4, 21.9%). The same was true in models for 8-isoprostane (ΣDEHP: 13.7%; 95% CI: 7.55, 20.1%; MBP: 37.3%; 95% CI: 27.6, 47.9%). Second, we examined the effect of including one predictor from each primary parent compound of interest in the same model (ΣDEHP, MBzP, MBP, MEP, and MCPP). In models of 8-OHdG, effect estimates were diminished compared with single-exposure models (e.g., 13.3%; 95% CI: 7.84, 19.1% for MBzP), and coefficients for ΣDEHP and MCPP lost statistical significance (see Supplemental Material, Table S4). In models of 8-isoprostane, effect estimates were also smaller in magnitude (e.g., 21.8%; 95% CI: 11.3, 33.2% for MBzP) but remained statistically significant for all phthalate metabolites (see Supplemental Material, Table S4).

## Discussion

We examined the association between repeated measures of urinary phthalate metabolites and 8-OHdG and 8-isoprostane as biomarkers of oxidative stress during pregnancy. We observed that all phthalate metabolites were associated with higher concentrations of both biomarkers. Associations were stronger with 8-isoprostane compared with 8-OHdG, and, among phthalates, MBzP, MBP, and MiBP showed strongest associations with both outcome measures.

Many different biomarkers have been used in environmental and other epidemiologic studies as a proxy of systemic levels of oxidative stress. These can have very different specificities, both in terms of mechanism (i.e., how they are produced) and downstream physiologic effect that the biomarkers themselves can have. The long-time goal of the National Institute of Environmental Health Sciences Biomarkers of Oxidative Stress Study has been to identify sensitive and specific markers of oxidative injury, as well as best methods for measuring these markers in animal and eventually human matrices (Kadiiska et al. 2013; National Institute of Environmental Health Sciences 2012). However, this task remains difficult, because of the numerous available markers and assays for detection, the long list of mechanisms that can cause oxidative stress, and the temporal instability of some biomarkers, among other reasons. We selected 8-OHdG and 8-isoprostane for measurement in this study because of their well-documented usefulness as systemic biomarkers of oxidative stress for establishing association with adverse health outcomes (Il’yasova et al. 2012), but also their representation of different cellular reactions to ROS exposure and the potential downstream effects of the biomarkers themselves.

Consistent with their low correlation in this and other studies (Stein et al. 2008), urinary levels of 8-OHdG and 8-isoprostane represent two distinct cellular processes. 8-OHdG is a DNA adduct formed in the presence of excess ROS (e.g., hydroxyl radicals) (Wu et al. 2004). Via repair mechanisms, oxidized nucleotides are excised from DNA and excreted in the urine, making repair capabilities an important factor in urinary concentrations (Wu et al. 2004). 8-isoprostane is formed in a non-enzymatic reaction between ROS and arachidonic acid and is advantageous because it is very specific to lipid oxidation, yet is not affected by dietary lipid intake, and is highly detectable in urine samples (Roberts and Morrow 2000). Measurement of specific isomers (e.g., the biologically active 8-iso-PGF_2α_) with liquid chromatography/mass spectrometry is preferable to enzyme immunoassay, although multiple isomers can be representative of oxidative lipid damage, and the liquid chromatography/mass spectrometry method in a study of this size is cost prohibitive (Il’yasova et al. 2012; Smith et al. 2011).

In addition to indicating systemic oxidative stress, these different products may be markers of or play a direct role in physiologic changes that have adverse consequences for pregnancy. Oxidative DNA damage, specifically indicated by 8-OHdG, occurring in the intrauterine compartment could result in apoptosis at the maternal–fetal interface (Heazell et al. 2007), which can lead to poor vascularization of the placenta and consequently preeclampsia and/or intrauterine growth restriction (Potdar et al. 2009). On the other hand, increased levels of prostaglandins such as 8-isoprostane may be particularly dangerous later in pregnancy because of their direct involvement in the preterm parturition pathway (Challis et al. 2009). This study illustrates that phthalates are associated with increases in both oxidative stress biomarkers in pregnant women, which suggests that exposure to phthalates could play a role in downstream pregnancy outcomes via multiple mechanisms.

In a number of *in vitro* studies, phthalates have been shown to cause increases in ROS and various markers of oxidative stress, potentially via activation of peroxisome proliferator–activated receptors or by increasing permeability of mitochondrial membranes (Hurst and Waxman 2003; Rosado-Berrios et al. 2011). These studies have been performed using DEHP and/or MEHP in a number of cell types, including placental cells (Tetz et al. 2013), Leydig cells (Erkekoglu et al. 2010; Zhou et al. 2013), neutrophils (Vetrano et al. 2010), and Kupffer cells (Rusyn et al. 2001). Other phthalates and their metabolites have been studied less frequently, although there is also evidence that they may be capable of inducing oxidative stress (O’Brien et al. 2001; Zhou et al. 2010).

In humans, studies of phthalates in relation to oxidative stress biomarkers have been limited. Two reports have examined the relationship among participants in the National Health and Nutrition Examination Survey, using gamma-glutamyl transferase and bilirubin in serum as markers of oxidative stress and a panel of phthalate metabolites similar to those measured in the present study. In the study of gamma-glutamyl transferase, positive associations were observed in association with MEHP only, although serum C-reactive protein, a systemic marker of inflammation that may also indicate oxidative stress, was positively associated with MiBP and MBzP (Ferguson et al. 2011). In the study of bilirubin, a potent antioxidant that may be inversely related to oxidative stress levels, DEHP and DBP metabolites as well as MCPP were found to be associated with significantly decreased bilirubin, although the strongest associations appeared to be for DEHP metabolites and MCPP (Ferguson et al. 2012).

Another cross-sectional study of urban-dwelling adults examined the relationship between phthalate metabolites and urinary MDA and 8-OHdG (*n* = 960) (Hong et al. 2009). The results from this analysis demonstrated significant and positive associations with DEHP metabolites as well as MBP and both oxidative stress biomarkers, although the positive associations with 8-OHdG lost significance in adjusted models. Regression coefficients were higher for MDA compared with 8-OHdG in DEHP metabolite models, but lower for MBP models. Regression coefficients were also higher for DEHP metabolites compared with MBP for both outcomes. Finally, one study in elderly subjects with measurements taken up to five times over 3 years observed a positive relationship between summed DEHP metabolites and urinary MDA levels (*n* = 560 subjects) (Kim et al. 2013). MBP levels were measured in the study but associations with MDA were not reported.

Our findings are somewhat consistent with these prior studies. As reported by Hong et al. (2009), we also observed positive associations between phthalates and 8-OHdG. Consistent with the studies of MDA, we observed higher levels of 8-isoprostane in association with urinary phthalate metabolites that appeared to be stronger than the associations observed with 8-OHdG. However, contrary to these studies, we observed the strongest associations for MBzP, MBP, and MiBP compared with DEHP metabolites for both outcome measures. This disparity could be attributable to differences in diet, product use, toxicant metabolism, and/or other differences between study populations. Considering our findings alone, the larger associations observed between MBzP, MBP, and MiBP with oxidative stress measures may be a result of lower temporal variability in those metabolites across pregnancy, which was observed in this study as well as others measuring phthalates at multiple time points during gestation (Adibi et al. 2008; Braun et al. 2012; Ferguson et al. 2014a). This would result in less measurement error and stronger associations, even if the true relationships between oxidative stress biomarkers and different phthalate metabolites were similar. Finally, it is possible that the associations observed are an effect of unknown confounders or other sources of error.

In conclusion, we report statistically significant increases in oxidative stress biomarkers in association with urinary phthalate metabolites during pregnancy. Our ability to detect these relationships may be attributable largely to our study design, with measurement of both urinary phthalate metabolites and oxidative stress biomarkers at up to four time points per subject across gestation. These associations with phthalate exposure may be important for pregnancy outcomes that are mediated by oxidative stress mechanisms. Additional exploration of these associations in other populations, particularly in nonpregnant women as well as men of reproductive age, children, and the elderly, may be of great importance for a range of other health outcomes that have been linked to phthalates in epidemiologic studies.

## Supplemental Material

(329 KB) PDF
