# Supplementary material for: Urinary Phthalate Metabolites and Biomarkers of Oxidative Stress in Pregnant Women: A Repeated Measures Analysis
Source: Environ Health Perspect. 2014 Nov 14;123(3):210–6. doi: 10.1289/ehp.1307996 (PMC4348741; doi:10.1289/ehp.1307996)
Supplement: (329 KB) PDF [file ehp.1307996.s001.508.pdf]

Environmental Health Perspectives doi:10.1289/ehp.1307996

Supplemental Material

Urinary Phthalate Metabolites and Biomarkers of Oxidative Stress in Pregnant Women: A Repeated Measures Analysis

Kelly K. Ferguson, Thomas F. McElrath, Yin-Hsiu Chen, Bhramar Mukherjee, and John D. Meeker

Table S1. Demographic characteristics in weighted study population including only subjects with complete covariates included in adjusted regression models[a] (N=464).

Characteristic	Percent
**Race/ethnicity (N=464)**	
White	60
African American	15
Other	25
**Education (N=464)**	
High school	14
Technical school	15
Junior college or some college	29
College graduate	41
**Health insurance (N=464)**	
Private/HMO/self-pay	82
Medicaid/SSI/MassHealth	18
**Body mass index at visit 1 (N=461)**	
<25 kg/m^2 (underweight to normal)	53
25-30 kg/m^2 (overweight)	27
≥30 kg/m^2 (obese to morbidly obese)	20
**Smoking during pregnancy (N=464)**	
Some	6
None	94
**Alcohol use during pregnancy (N=460)**	
Some	5
None	95
**Parity (N=464)**	
Nulliparous	46
Parous	54

HMO, health maintenance organization, SSI, supplemental security income. Distributions of demographic characteristics created from inverse probability weightings for case-control status.

[a]Covariates included in final statistical models were not the same as those described in Table S1. Covariates for final models included: urinary specific gravity, gestational age at sample collection, race/ethnicity, education level, health insurance provider, body mass index (time-varying), time of day of urine sample collection, and parity of infant.

Table S2. Urinary phthalate metabolite concentrations (geometric mean and geometric standard deviation) by categorical demographic characteristics in all samples measured from weighted population (N=1678 samples, N=482 subjects).

**Characteristic**	**MEHHP (µg/L)**	**MEOHP (µg/L)**	**MECPP (µg/L)**	**∑DEHP (µmol/L)**	**MBzP (µg/L)**	**MiBP (µg/L)**	**MEP (µg/L)**	**MCPP (µg/L)**
**Race/ethnicity**								
White (Ref.)	35.8 (2.11)	18.9 (2.03)	43.9 (2.02)	0.39 (1.91)	5.67 (1.63)	6.28 (1.33)	111 (3.06)	2.00 (1.78)
African-American	35.8 (2.13)	18.4 (2.16)	42.8 (2.22)	0.40 (2.05)	10.5 (1.90)*	11.2 (1.36)*	285 (3.04)*	2.49 (2.38)
Other	29.9 (2.11)	16.9 (2.06)	42.9 (2.27)	0.37 (1.98)	9.31 (2.15)*	9.44 (1.47)*	165 (3.26)*	2.13 (2.02)
**Education**								
High school (Ref.)	27.8 (1.76)	15.2 (1.79)	37.0 (1.94)	0.32 (1.74)	15.6 (2.30)	10.7 (1.25)	286 (3.22)	2.52 (2.04)
Technical school	30.0 (2.10)	15.5 (2.07)	34.7 (2.11)	0.32 (1.98)	8.18 (1.97)*	9.45 (1.44)	197 (3.01)	2.10 (1.90)
Junior college or some college	33.2 (2.29)	17.9 (2.21)	44.1 (2.23)	0.38 (2.01)	6.07 (1.72)*	6.43 (1.58)*	133 (3.10)*	1.99 (1.98)
College graduate	38.7 (2.10)	20.9 (2.00)	49.5 (2.06)	0.44 (1.93)	5.73 (1.59)*	7.05 (1.30)*	101 (3.04)*	2.06 (1.88)
**Health insurance**								
Private insurance/HMO/Self-pay (Ref.)	34.6 (2.21)	18.7 (2.13)	45.0 (2.18)	0.40 (2.00)	5.93 (1.66)	6.99 (1.43)	120 (3.20)	2.02 (1.92)
Medicaid/SSI/MassHealth	30.7 (1.76)	16.1 (1.78)	36.4 (1.85)	0.33 (1.73)	15.9 (2.12)*	11.4 (1.24)*	295 (2.79)*	2.39 (1.75)
**BMI at visit 1**								
Less than 25 kg/m^2 (Ref.)	33.7 (2.09)	18.3 (1.99)	43.5 (2.14)	0.38 (1.95)	6.58 (1.86)	7.08 (1.30)	116 (2.90)	1.95 (1.82)
25 to less than 30 kg/m^2	34.0 (2.34)	17.7 (2.34)	41.4 (2.19)	0.38 (2.04)	6.08 (1.69)	7.89 (1.56)	162 (3.96)*	2.19 (2.06)
Greater than 30 kg/m^2	36.3 (1.94)	19.4 (1.90)	46.8 (1.96)	0.41 (1.82)	10.1 (1.88)*	8.88 (1.51)*	202 (3.18)*	2.38 (1.97)
**Tobacco use**								
Smoked during pregnancy (Ref.)	38.4 (1.71)	19.6 (1.65)	46.9 (1.64)	0.40 (1.64)	15.4 (1.75)*	9.06 (1.24)	177 (3.94)	3.55 (1.94)
No smoking during pregnancy	33.7 (2.15)	18.1 (2.08)	43.2 (2.15)	0.38 (1.97)	6.76 (1.83)	7.56 (1.42)	140 (3.26)	2.03 (1.91)*
**Alcohol use**								
Alcohol use during pregnancy (Ref.)	58.3 (1.84)	31.2 (1.78)	81.1 (2.05)	0.65 (1.87)	5.33 (1.41)	8.38 (1.53)	138 (2.88)	2.79 (2.72)
No alcohol use during pregnancy	32.9 (2.11)*	17.6 (2.05)*	41.8 (2.09)*	0.37 (1.93)	7.23 (1.89)*	7.61 (1.41)	143 (3.32)	2.07 (1.89)
**Parity**	36.4 (2.14)	19.7 (2.08)	46.3 (2.13)	0.41 (1.96)	6.21 (1.79)	7.26 (1.43)	149 (3.16)	2.04 (1.90)
Nulliparous (Ref.)								
Non-nulliparous	32.4 (2.10)	17.2 (2.03)	41.3 (2.09)	0.36 (1.93)	7.87 (1.89)*	7.91 (1.39)	136 (3.40)	2.15 (1.95)

Ref., reference category. HMO, Health Maintenance Organization. SSI, Supplemental Security Income. BMI, body mass index.

*$p<0.05$ for significant difference in biomarker concentration from reference category, estimated from weighted linear mixed model with random intercepts for subject ID. All biomarkers adjusted for urinary specific gravity.

Table S3. Percent difference (95% confidence intervals) in oxidative stress biomarker in association with interquartile range increase in phthalate metabolite level. Estimates from adjusted[a] linear mixed effect models with random intercepts for subject ID, including cases (N=405 samples, N=127 subjects) or controls only (N=1199 samples, N=337 subjects).

Metabolite	Cases 8-OHdG % difference (95% CI)	p	Controls 8-OHdG % difference (95% CI)	p	Cases 8-Isoprostane % difference (95% CI)	p	Controls 8-Isoprostane % difference (95% CI)	p
MEHP	4.11 (-2.13, 10.8)	0.202	2.77 (-0.47, 6.11)	0.10	11.8 (1.27, 23.3)	0.028	14.3 (8.15, 20.7)	<0.001
MEHHP	6.90 (0.02, 14.3)	0.050	8.07 (4.74, 11.5)	<0.001	11.8 (0.69, 24.1)	0.038	15.2 (9.15, 21.5)	<0.001
MEOHP	6.22 (-0.33, 13.2)	0.064	7.15 (3.91, 10.5)	<0.001	10.9 (0.44, 22.5)	0.042	15.5 (9.61, 21.7)	<0.001
MECPP	6.14 (-0.76, 13.5)	0.083	4.90 (2.23, 7.64)	<0.001	14.8 (3.02, 28.0)	0.013	16.9 (11.9, 22.2)	<0.001
∑DEHP	6.08 (-0.61, 13.2)	0.077	6.65 (3.22, 10.2)	<0.001	12.9 (1.92, 25.0)	0.021	19.1 (12.6, 25.9)	<0.001
MBzP	18.5 (7.60, 30.5)	0.001	19.5 (14.6, 24.5)	<0.001	50.3 (27.4, 77.4)	<0.001	39.8 (29.8, 50.6)	<0.001
MBP	8.92 (2.03, 16.3)	0.011	17.5 (13.1, 22.2)	<0.001	30.9 (15.8, 48.0)	<0.001	40.7 (31.0, 51.0)	<0.001
MiBP	20.3 (10.2, 31.3)	<0.001	30.3 (24.4, 36.5)	<0.001	52.5 (32.2, 76.0)	<0.001	56.4 (43.9, 69.9)	<0.001
MEP	19.2 (10.3, 28.8)	<0.001	11.2 (7.12, 15.4)	<0.001	15.2 (0.70, 31.8)	0.040	19.1 (11.5, 27.3)	<0.001
MCPP	5.28 (-0.73, 11.6)	0.087	7.11 (3.77, 10.6)	<0.001	19.2 (8.46, 31.1)	<0.001	19.9 (13.5, 26.6)	<0.001

8-OHdG, 8-hydroxydeoxyguanosine.[a]Adjusted for urinary specific gravity, gestational age at sample collection, race/ethnicity, education level, health insurance provider, body mass index (time-varying), time of day of urine sample collection (before vs. after 1pm, time-varying), and parity of infant.

Table S4. Percent difference (95% confidence intervals) in oxidative stress biomarker in association with interquartile range increase in phthalate metabolite level. All metabolites were included as predictors of one oxidative stress biomarker in each model. Estimates from adjusted[a] linear mixed effect models with random intercepts for subject ID (N=1604 samples, N=464 subjects).

Metabolite	8-OHdG % difference (95% CI)	p	8-Isoprostane % difference (95% CI)	p
∑DEHP	2.98 (-0.53, 6.60)	0.10	9.49 (3.25, 16.1)	0.003
MBzP	13.3 (7.84, 19.1)	<0.001	21.8 (11.3, 33.2)	<0.001
MBP	9.08 (4.08, 14.3)	<0.001	20.4 (10.5, 31.2)	<0.001
MEP	8.46 (4.41, 12.7)	<0.001	12.0 (4.71, 19.7)	0.001
MCPP	1.60 (-1.92, 5.24)	0.38	7.75 (1.47, 14.4)	0.015

8-OHdG, 8-hydroxydeoxyguanosine.[a]Adjusted for urinary specific gravity, gestational age at sample collection, race/ethnicity, education level, health insurance provider, body mass index (time-varying), time of day of urine sample collection (before vs. after 1pm, time-varying), and parity of infant. Models include inverse probability weights to adjust for case-control study design.
